# Flying at No Mechanical Energy Cost: Disclosing the Secret of Wandering Albatrosses

**DOI:** 10.1371/journal.pone.0041449

**Published:** 2012-09-05

**Authors:** Gottfried Sachs, Johannes Traugott, Anna P. Nesterova, Giacomo Dell'Omo, Franz Kümmeth, Wolfgang Heidrich, Alexei L. Vyssotski, Francesco Bonadonna

**Affiliations:** 1 Institute of Flight System Dynamics, Technische Universität München, Garching, Germany; 2 Behavioural Ecology Group, Centre d'Ecologie Fonctionnelle et Evolutive, U.M.R., 5175 CNRS, Montpellier, France; 3 Ornis italica, Rome, Italy; 4 e-obs GmbH, Grünwald, Germany; 5 Institute of Neuroinformatics, University of Zurich/ETH Zurich, Zurich, Switzerland; Phillip Island Nature Parks, Australia

## Abstract

Albatrosses do something that no other birds are able to do: fly thousands of kilometres at no mechanical cost. This is possible because they use dynamic soaring, a flight mode that enables them to gain the energy required for flying from wind. Until now, the physical mechanisms of the energy gain in terms of the energy transfer from the wind to the bird were mostly unknown. Here we show that the energy gain is achieved by a dynamic flight manoeuvre consisting of a continually repeated up-down curve with optimal adjustment to the wind. We determined the energy obtained from the wind by analysing the measured trajectories of free flying birds using a new GPS-signal tracking method yielding a high precision. Our results reveal an evolutionary adaptation to an extreme environment, and may support recent biologically inspired research on robotic aircraft that might utilize albatrosses' flight technique for engineless propulsion.

## Introduction

The flight of birds has always fascinated humans. Leonardo da Vinci was one of the first to bring imagination to reality by studying bird flight and drawing flying machines. Now humans can fly. However, not all the secrets of flight have been discovered, and we can still learn from birds. For example, albatrosses (procellariiformes) seem to fly at almost no cost and can cover huge distances during their foraging trips without flapping their wings [1]. These long-living birds spend most of their lives in flight over the sea and return to small oceanic islands only for breeding [2]. As foraging grounds of all albatross species are pelagic, they have to find productive areas repetitively during the breeding period. Trips of 15,200 km or flights around the world in 46 days have been reported for wandering albatrosses (*Diomedea exulans*) [3], [4]. Moreover, albatrosses are capable of mean ground speeds higher than 127 km/h and can maintain such speeds for more than 8 hours [5].

From the aerodynamic properties of albatrosses [6], one can estimate the power required for flying. Assuming a maximum lift-to-drag ratio of 20 and 8.5 kg weight [6], an albatross has to develop a power of 81.0 W for flying at 70 km/h. A gasoline engine producing such power would consume about 0.9 litre of fuel per day (see Text S1). Thus, substantial energy is needed to keep a bird flying. A lighter and slower bird than wandering albatross, the pink footed geese (*Anser brachyrhynchus*), loses 0.34 g/km during flying [7]. At this geese's rate, an albatross performing a 15,200 km flight would lose half of its body mass, which is between 8 and 11 kg for a male [8]. This implies that an albatross should eat enough prey to replace the lost weight, and make enough reserves to keep its eggs warm during 10 to 20 days after returning from a foraging trip.

Given these calculations, it would be difficult to explain albatrosses' foraging strategies without considering proper adaptations necessary to reduce energy expenditures. The first of these adaptations is anatomical. Albatrosses have an elbow-lock system operating which keeps their wings open without any muscle activity, e.g. no energy expenditure [9], [10]. The second adaptation concerns a flight mode – dynamic soaring [5]. It enables albatrosses to gain the energy required for flying from the constant strong winds common in the marine environment [1]. What physical rules in terms of special flight technique or interaction between bird's motion and wind govern such outstanding travelling performance in regard to the energy cost of flying?

It is important to distinguish between the large-scale movement that appears as a steady-state cruise of long-distance travel and the small-scale movements that are flight manoeuvres of highly dynamic nature. The large-scale movements which are of the order of hundreds to thousands of kilometres are well documented [2], [3], [4], [5], [11]. However, the small-scale movements being of the order of tens to hundreds of metres and constituting dynamic soaring [6] have not yet been experimentally investigated. This is because up to now there were no tracking devices providing the necessary high resolution (in both position and sampling rate) and no adequate mathematical method for computing the flight path with the required high precision. As result, we still do not know which physical mechanism of energy extraction from the wind allows albatrosses to fly at no mechanical cost.

There are different theories of the small-scale movements in albatrosses [11], [12], [13], [14], [15], [16], [17]. The theory of wind-gradient soaring is based on gradient in the shear wind gradient above the sea surface [11], [12], [13], [14]. Another theory is related to gust soaring which explains discontinuities in the wind flow [15], [16], [17].

This paper presents the first experimental results on the small-scale movements constituting dynamics soaring which enables albatrosses to fly at no mechanical energy cost. We use in-flight-measurement data to show how birds gain the energy required for flying without flapping their wings from the moving air in the shear wind above the sea surface. Based on these high-precision data achieved with a new, in-house developed GPS-signal tracking method, it was possible to analyse the dynamic soaring flight manoeuvre in detail.

## Results and Discussion

### Experimental results on dynamic soaring

**Figure 1:**
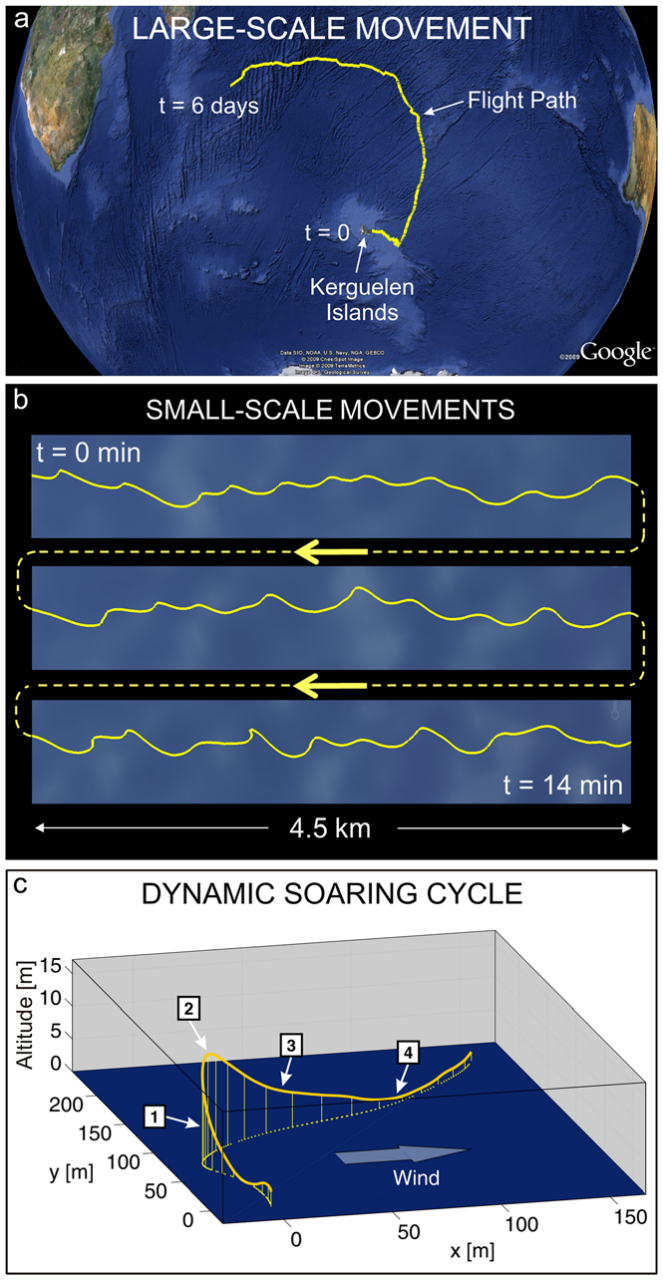
Large- and small-scale movements and dynamic soaring cycle. (**a**) Large -scale movement. The 4850 km path (projected to the sea surface) of a long-distance flight of a wandering albatross is shown. Logging stopped after the first 6.0 days of this 30-day-long foraging trip. (**b**) Small-scale movements. A 14 min portion of the long-distance flight from Fig. 1a shows a sequence of three connected parts. The flight path consists entirely of winding and curving segments, not exhibiting any straight horizontal sections. (**c**) Dynamic soaring cycle. The small-scale movements consisted of dynamics soaring cycles featuring distinct motions in the longitudinal, lateral, and vertical directions. Each dynamic soaring cycle consists of (1) a windward climb, (2) a curve from wind- to leeward at the upper altitude, (3) a leeward descent and (4) a curve from lee- to windward at low altitude, close to the sea surface.

In the longest track the GPS recorded 4,850 km during the first six days of a total 30-day foraging trip (Fig. 1a). While showing a large distance covered on a global scale, this trajectory does not provide any information about the small-scale flight manoeuvres involved in the whole flight. It is only at small scale resolution (Fig. 1b) that the typical pattern of dynamic soaring becomes visible. The whole flight consists of curved trajectory segments that are continuously repeated close to the water surface. A visual inspection of all our records (based on the coarse 1 Hz online solution) revealed that birds never fly in straight lines and are always confined to a low altitude region.

Analysing further details of the individual flight cycles required an even smaller scale (Fig. 1c). Whereas no special GPS raw data analysis was necessary for previous plots (Fig. 1a.b), bird's position here was calculated using fine-scale 10 Hz position fixes derived from a GPS post-processing software. According to the built-in error estimator of our program, we could identify the total position error with respect to the starting point of the cycle to stay below 4 dm. One recognizes immediately that the manoeuvre was made up of horizontal curve phases superimposed by climbing and descending phases. With regard to the local wind direction, the manoeuvre could be partitioned into four flight phases which are representative for each cycle: (1) a windward climb, (2) the upper curve from wind- to leeward flight direction, (3) a leeward descent, and finally (4) a lower curve from lee- to windward flight. We considered such flight cycle as the essential key to understanding how the birds extract energy from the wind – this cycle is the fundamental element of dynamic soaring.

**Figure 2:**
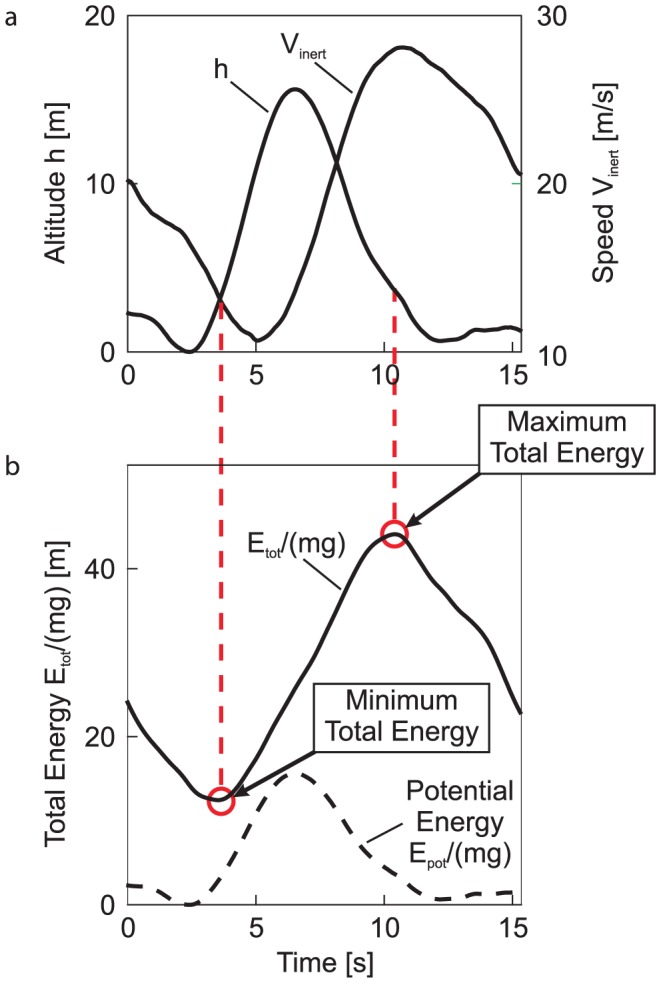
Dynamic soaring cycle (same cycle as Fig. 1c). (**a**) Altitude 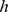 and inertial speed 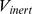. The altitude shows a cyclic behaviour (between lowest point near to the sea surface and top of trajectory). The speed, which is also cyclic, follows the altitude with a time lag. Speed starts to increase during the climbing phase, despite an increase in altitude. This indicates that there is a simultaneous increase of potential and kinetic energy to yield an increase of the total energy. The altitude 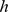 is affected with an estimated error drift of 2 cm/s yielding a maximum bias of 31 cm after 15 s. 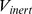 is biased by 2.5 cm/s (0.1–0.2% relative error). (**b**) Total energy 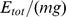 and potential energy 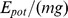. The total energy, 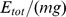, presented in form of a solid line has cyclic characteristics, too. It begins to increase during the windward climb and continues to do so until the peak of the trajectory has been passed. The maximum value of the total energy is reached during the leeward descent. This is indicated by a red circle and a dashed line linking Figs. 2a and 2b. There is a large energy gain (∼360% relative to the beginning of the dynamic soaring cycle). The total energy curve is smooth and continuous. As a consequence, the extraction of energy from the shear wind is also smooth and continuous, without any discontinuities or energy pulses. Furthermore, the energy gain is achieved not at the low level, but in the upper part of the altitude region, around the top of the trajectory. The bias of 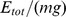 is estimated to 0.2–0.5%. The potential energy, 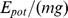, presented in form of a dashed line is considerably smaller than the total energy. Thus, the kinetic energy given by the difference between the solid and dashed lines exceeds significantly the potential energy. This holds particularly for the phase of the energy gain from the wind. In the second part of that phase, the potential energy is even decreasing to reach again its lowest level.

A closer examination of the cycle's quantities related to dynamic soaring was intended to expose the physics of the manoeuvre (Fig. 2a). The time of the cycle was in the order of 15 s. The altitude region extends from approximately 0 to 15 m. In this region, the wind speed increases from zero to values approaching the free airflow [18], and the bird's speed varies between about 10 and nearly 30 m/s.

An interactive visualization of the discussed 3-dimensional dynamic soaring trajectory is provided in Text S2.

The results of an analysis of the bird's total energy state during the manoeuvre are presented in Fig. 2b (solid line), which shows the time history of
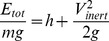
where 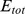 is the total energy in terms of the sum of potential and kinetic energy, 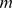 is the mass of the bird, 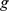 is the acceleration due to gravity, 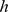 is the altitude, and 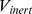 is the speed with respect to the Earth (which is used as an inertial reference system). Dividing by *mg* yields the specific total energy which does not require any assumptions regarding the bird's mass.

The energy transfer has the following characteristics. The total energy begins to increase during the windward climb. At the top of the trajectory, the energy gain does not come to a stop, but continues to increase. The total energy finally reaches its maximum value during the leeward descent, after the bird has already started to loose altitude. This is indicated by the red circles in Fig. 2b and the dashed lines linking Figs. 2b and 2a. These dashed lines establish a direct relation between the total energy and the motion quantities (altitude, speed). As a result, energy gain as high as 360% is achieved in relation to the starting point of the cycle. After having reached its maximum, the total energy decreases in the last part of the descent.

Furthermore, Fig. 2b also presents the time history of the potential energy related to the weight (dashed line). The difference between the solid and dashed lines yields the kinetic energy. Since there is a large difference, the total energy is primarily made up of the kinetic energy whereas the potential energy remains on a significantly lower level throughout the entire dynamic soaring cycle. In particular, the gain in the total energy gain is predominantly due to an increase of the kinetic energy.

### Upper curve: characteristic flight phase of dynamic soaring

**Figure 3:**
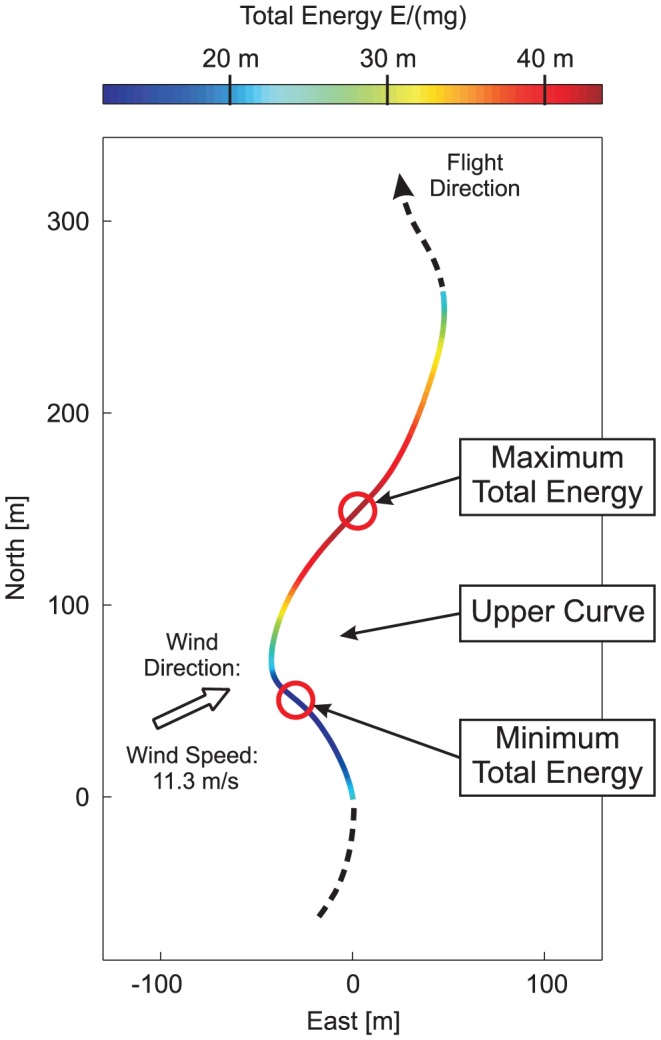
Total energy mapped to flight trajectory (for the cycle from **Fig. 1c**). The total energy, 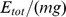, is indicated by colour coding. Quantification is possible with reference to the bar (at the top) which establishes a relation between colour and total energy. The energy gain is achieved in the part of the curve where the colour changes from blue to red. The total energy reaches a maximum at the end of the upper curve, after the bird has changed its flight direction from wind- to leeward (dark-red colouring). Thereafter, the total energy begins to decrease and reaches a minimum past the lower curve where the bird has changed its flight direction from lee- to windward. Both the minimum and the maximum total energy states correspond with the ones in Figs. 2a and 2b (as indicated by red cycles in each Fig.). The coloured part of the trajectory coincides with the time span of the diagrams in Fig. 2. The depicted trajectory part shows an error drift of 1.5 cm/s yielding an estimated bias of 23 cm at the end of the cycle. The direction and the speed of the wind are also indicated. The wind speed holds for 10 m altitude.

To understand the energy gain mechanisms in dynamic soaring it is essential to examine the trajectory section where this gain is achieved (Fig. 3). The energy gain is achieved in the part of the curve where the flight direction changes from windward to leeward. Both the minimum and the maximum total energy states are highlighted by red circles. Note that these circles correspond with the ones in Figs. 2a and 2b as well as with the altitude values marked by red lines. Consequently, the energy gain takes place in the upper curve located between the circles. As a result, the upper curve can be identified as the characteristic flight phase of dynamic soaring where the energy gain is achieved.

The physical mechanism of the energy gain is the propulsive effect which the wind exerts. This mechanism can be understood as an effect in which the bird is taken by the wind. Thus the inertial speed increases when the bird changes its flight direction from windward to leeward in the upper curve.

This gain is opposed by an energy loss in the lower curve of the manoeuvre with a change of flight direction from lee- to windward (red- to blue change of colour in Fig 3.). Because the wind speed is small at low altitudes, the inevitable energy loss does not make up for the energy gain at high altitudes but yields a net surplus sufficient to compensate for the dissipative drag effect.

**Figure 4:**
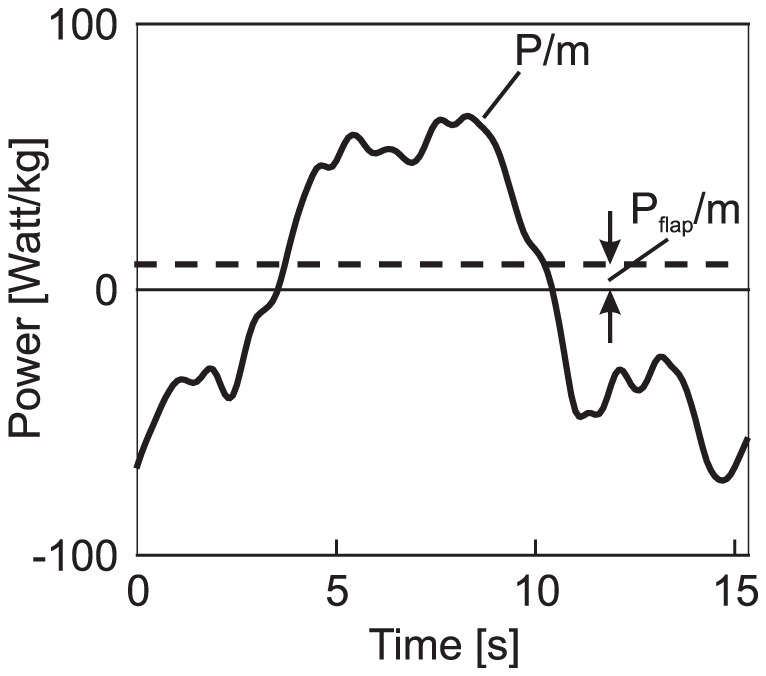
Mechanical power balance during dynamic soaring cycle (for the same cycle as in **Fig. 1c**). The mechanical power in terms of the surplus of the power obtained from the wind over the dissipative drag effect is presented. The mechanical power related to the mass, 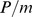 (solid line), can be divided mainly into two parts: A part showing a high positive power level which is correlated with the upper altitude region where the energy gain from the wind is achieved. This part is opposed by a negative power level which is correlated with the flight phase close to the water surface where the energy loss occurs. The level of negative power is smaller than the one of positive power. The resulting integral power surplus is just sufficient to compensate for the dissipative drag effect. The data of 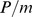 are virtually unbiased. The mechanical power available in flapping flight, 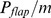 (dashed line), is shown for comparison purposes. It is based on the albatross power data described in the Introduction section (maximum lift-to-drag ratio of 20, 8.5 kg mass, and 70 km/h speed). 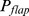 is much smaller in magnitude than 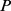. This holds for both the positive and the negative parts of 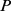.

The understanding of the energy gain mechanism can be improved by an analysis of the power characteristics (Fig. 4). The power is at a high level throughout the whole energy gain phase, yielding a maximum value of
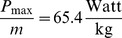
For comparison purposes, the mechanical power available in flapping flight is also shown in Fig. 3, denoted by 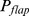, with using 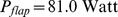 as reference. Comparison of both quantities in the energy gain phase shows that 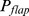 is much smaller than 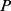. This means that the large 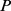 values in the energy gain phase cannot be due to flapping the wings. Rather, there is another mechanism which is the effect of the wind in the upper curve.

Analogue considerations hold for the part where 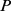 is negative, relating to the lower curve (Fig. 4). Firstly, the fact that there are negative power values  excludes that there is wing flapping. Secondly, the negative 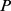 values are so large in magnitude that they cannot be caused by the aerodynamic drag because this force is too small. Thus, the negative values are primarily due to the wind in the lower curve where the bird changes its flight direction from leeward to windward (Fig. 3).

As a conclusion, the power characteristics in terms of both the large positive and negative power values cannot be explained by wing flapping. Rather, there is another more powerful mechanism which is the interaction between the wind and the motion of the bird.

Dynamic soaring and the energy gain mechanism could be best explained by focusing on a single dynamic soaring cycle. Supplementary, more dynamic soaring cycles are presented in Figs. S1, S2, and S3, showing that there is repeatability in all essential features concerning the total energy transfer mechanism from the wind to the bird. This energy transfer particularly holds for the upper altitude region in terms of the upper curve where the energy gain is achieved. Furthermore, the mechanical power in the energy gain phase is always much larger than the maximum power possible by flapping the wings. This can be also evidence that the energy gain cannot be generated by flapping the wings, but is created by the wind in the upper curve.

### Resolving the controversies in theories of dynamic soaring

**Figure 5:**
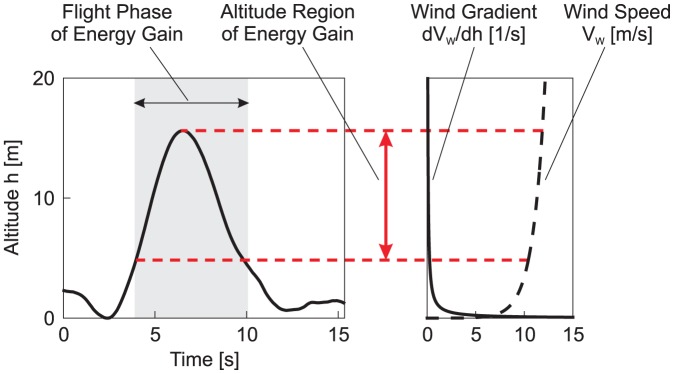
Relationship between energy gain, altitude and wind gradient. The left and right diagrams show the relationship between the shear wind layer above the sea surface and the altitude region where the energy gain from the wind is achieved (during the dynamic soaring cycle shown in Fig. 2a and 2b). On the left diagram, the energy gain phase is indicated by grey shading. This corresponds with the phase between the minimum and maximum of the total energy shown in Fig. 2a and 2b by red circles. The right diagram in Fig. 4 shows the wind speed (dashed line) and the wind gradient (solid line) as functions of altitude. The wind speed at 10 m altitude was determined to yield 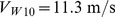. The shear wind profile, 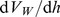, is based on a logarithmic wind model [18]. The altitude region where the energy gain is achieved is indicated by red dashed lines which establish a link between the left and right diagrams. At small altitudes, large changes in the wind speed occur, resulting in a high wind gradient. As the altitude increases, the changes in the wind speed continually decrease to become very small in the altitude region where the energy gain is achieved. As a result, the wind gradient is very weak at this stage.

We performed an analysis showing the interrelation between the shear wind region encountered close to the sea surface and the bird's total energy state. The results of this analysis are presented in Fig. 5 which shows the altitude profile of the described dynamic soaring cycle on the left panel and, on the right panel, both the wind speed, 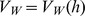 and the wind gradient, 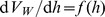, as functions of altitude. At low altitudes there are major changes in the wind speed corresponding to a high wind gradient. However, at an elevated altitude level, which is the region where the actual energy gain is achieved (indicated by red dashed lines in the figure), the wind speed shows only little change corresponding to very small wind gradient 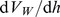. With regard to the theory of wind-gradient soaring, this means that the wind gradient itself is insignificant for the gain of energy. The energy gain is rather due to the change in flight direction from windward to leeward in the upper curve.

The total energy plot (Fig. 2b) shows a smooth behaviour without any discontinuities. With regard to the theory of gust soaring, this means that there are no discontinuities or energy pulses. Instead, there is a continuous extraction of energy from the shear wind to the bird. Furthermore, the energy gain is not attained during flight close to the water surface. Rather, it is achieved in the upper altitude region, around the top of the trajectory, due to the change in the flight direction from windward to leeward.

### Future directions

The energy gain mechanism in albatrosses presented in this paper has potential to be used as a guide for engineers who draw inspiration from animal behaviour for the design of aerial vehicles. Evidence for this perspective is that biologically inspired research and development activities currently take place in aeronautics [16], [19], [20], [21], [22], [23]. The physical mechanism used by albatrosses for flying at no mechanical energy cost can inspire further technical thinking to make aircraft more energy-efficient.

## Methods

### Subjects and experimental protocols

The field tests took place at the Cap Ratmanoff (Kerguelen archipelago) breeding colony of wandering albatrosses (*Diomedea exulans*) during a field campaign of three months. Twenty albatrosses were equipped with GPS logging units (details below). The devices were attached to the birds' back feathers by Tesa tape (number 4651, TESA s.a.s. France) following a standard procedure proposed by Wilson and co-workers [24]. Individuals were equipped during incubation shifts, shortly before leaving for a foraging trip. The birds were sexed based on the colour and shape of the beak and head [8]. We did not measure the body mass because this was not necessary for achieving the results presented in our paper, and weighting procedure may be stressing for wandering albatrosses (FB personal observation). However, all birds were adult breeding birds whose body mass reported in literature is 9770±875 g for males and 7690±559 g for females. The loggers weighted from 1 to 1.4% (with 103 g in the heaviest version of the logger) of the birds' body weight, and were well below the maximum load recommended by Philips and co-workers (3%) [25]. After recovering the devices from homed birds, 16 loggers carried valid high-resolution flight recordings (80% success rate). In total, we obtained 10 Hz raw data recordings during 36 foraging days. Logging time and foraging trip duration varied in time (table S1 and figure S4); the longest recorded track is shown in Fig. 1a.

### Hardware

We used miniaturized single-frequency GPS loggers (by e-obs GmbH, Germany, and Technosmart, Italy). They all exhibited the common key feature of recording not only position fixes but also GPS raw data. Raw data carry all information required for position recalculation back in the office. The Ublox LEA-4T GPS module, which constitutes the backbone of our loggers, makes the raw data accessible with a sampling rate as high as 10 Hz whereas the maximum nominal output rate of the position fixes calculated online by the module's CPU is 4 Hz. As we aimed to log both raw data and online solution simultaneously, we limited the sampling rate to 1 Hz only for position fixes (test confirmed this configuration to work reliably.) The resulting data stream amounted to approximately 9.6 MB/h for the 10 Hz raw data and 0.2 MB/h for the 1 Hz online solution. All logger types featured 8 MB internal flash memory and two models used a 2 GB SD-card as memory extension. High performance 3.6 V primary Li-SOCl2 cells served as power supply.

### GPS data processing

Besides other factors such as atmospheric conditions, signal shadowing and hardware characteristics, the accuracy achievable with GPS depends heavily on the algorithms necessary for transforming receiver measurements of satellite-emitted signals (the “raw-data”) to position and velocity. We developed specialized raw data post-processing software to increase positioning accuracy from the meter range (which is a typical value for the coarse but robust online solution of single-frequency receivers) to the decimetre range, depending on environmental conditions. In addition to increase precision, the software takes full advantage of the high rate (10 Hz) available in the raw data. The core algorithm of our program consists of a novel L1 time-differential carrier-phase-based processing engine [26], [27]. Carrier phase measurements are very precise observations but they are inevitably affected by an ambiguity (i.e. measurements are biased by an unknown multiple of the carrier signal wave length). They are sensitive to disturbances such as signal shadowing and reflections. Consequently, carrier phase measurements are generally difficult to handle. Therefore, other high-precision techniques using these measurements typically have to fall back to differential corrections recorded by a near-by base-station (differential GPS), and (mostly static) initialization. In the scope of the Albatross project this would have been very difficult to achieve in the field if ever possible at all. Our software works without these additional aids. The drawback of this advantage is a residual error drift affecting the final solution. This limits the time of precisely measurable trajectories to intervals ranging from 30 seconds to up to 5 minutes, depending on environmental conditions. Furthermore, the achieved precision holds for the trajectory relative to its trajectory's starting point, which may be biased by several metres.

Operating on the back of an Albatross is a challenging task for any kind of GPS receiver. The bird is flying very close to an agitated water-surface, which is likely to cause severe and unpredictable signal reflections. Moreover, the bird is constantly manoeuvring, causing frequent loss-of-signal-lock due to antenna tilting and signal shadowing. Although these are very difficult environmental conditions for our software to operate, we succeeded to process selected trajectory intervals based on precise carrier-phase recordings.

In conclusion, the novel GPS-signal tracking method developed in-house improves the precision dramatically from the meter range (typical for common animal-tracking GPS receivers [28]) to the decimetre range.

### Wind

Wind information was obtained from SeaWinds on QuikSCAT Level 3 Daily, Gridded Ocean Wind Vectors (JPL SeaWinds Project [29]). The data are sampled on an approximately 0.25°×0.25° global grid twice a day (equal to 28 km in N-S and 18 km in E-W direction at 49°S). The data provide local wind velocity vectors at a reference altitude of 10 m with an accuracy of 2 m/s (or 10% for velocities above 20 m/s) and the wind direction within +/−20°. For calculating the wind at the respective trajectory point we used one-dimensional linear interpolation in the time domain and bivariate Akima interpolation in the position domain, as H. Müller showed in his “term paper” QuikSCAT L3 Global Wind Data – Projection on Local Flight Trajectories and 3D Interactive Visualization (this document is availlable at the Institute of Flight System Dynamics, Technische Universität München).

## Supporting Information

Figure S1
**Dynamic soaring cycle 1.** (**a**) Altitude 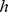 and inertial speed 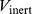. The behaviour of the altitude and the speed correspond with that of Fig. 2 of the main manuscript, showing the repeatability in the flight pattern. This particularly holds for the cyclic characteristics of both quantities, for their magnitude during the dynamic soaring cycle and for the time lag of the speed relative to the altitude. (**b**) Total energy 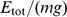 and mechanical power 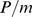 and 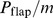. The behaviour of the total energy also corresponds to Fig. 2. In particular, this holds for the energy gain phase which is associated with the upper altitude region. Furthermore, there is a large energy gain of about 350% relative to the beginning of the dynamic soaring cycle, similar to Fig. 2. As a result, the total energy characteristics confirm the repeatability in the energy gain mechanism in terms of an energy transfer from the wind to the bird. The mechanical power, (solid line), shows two parts as a characteristic feature of dynamic soaring: A part with a high positive power level, associated with the upper altitude region where the energy gain from the wind is achieved. The other part shows a negative power level associated with the lower altitude region where the energy loss occurs. The mechanical power level available in flapping flight (dashed line) is much smaller in magnitude than during of the dynamic soaring cycle. As a result, the large power values cannot be generated by flapping the wings. Instead, this is due to the effect of the wind in terms of the interaction between the moving air and the motion of the bird.(EPS)

Figure S2
**Dynamic soaring cycle 2.** (**a**) Altitude 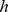 and inertial speed 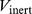, (**b**) Total energy 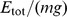 and mechanical power 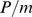 and 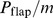, In Figure S2, the same characteristics hold as in Figure S1.(EPS)

Figure S3
**Dynamic soaring cycle 3.** (**a**) Altitude 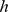 and inertial speed 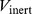, (**b**) Total energy 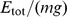 and mechanical power 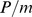 and 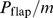, In Figure S3, the same characteristics hold as in Figure S1.(EPS)

Figure S4 **Albatrosses' long-distance flights.** Individual paths (projected to the sea surface) of long-distance flights of 11 albatrosses are represented. Both males (1 m, 3 m, 6 m) and females (2f, 6f, 7f, 14f, 91f, 94f, 901f, 1201f) were tracked. Complete paths were recorded for 1 m, 6 m, and 7f individuals and information on the part of the paths is available for the other birds. The scope of the study was to obtain high resolution data, and not necessarily complete paths. Additional information on 14f path that was longer than others is provided in Fig. 1a. The recording times for the other five birds are short, and their paths are not presented. The image was generated in the Google Earth Pro software.
(TIF)

Text S1 **Calculation of fuel consumption of a gasoline engine producing the same power as a wandering albatross.**
(DOCX)

Text S2 **Interactive 3-dimensional visualization of dynamic soaring.**
(DOCX)

Table S1 **Details on the recorded long-distance flights of tracked wandering albatrosses are given (see also Figure S4).** The trip parameters presented were calculated based on segment of the trip for which GPS data were available. Travel speed reflects overall speed including flying and resting on the water surface.
(DOCX)
