# Supplementary figures and images for: Flying at No Mechanical Energy Cost: Disclosing the Secret of Wandering Albatrosses
Source: PLoS One. 2012 Sep 5;7(9):e41449. doi: 10.1371/journal.pone.0041449 (PMC3434196; doi:10.1371/journal.pone.0041449)

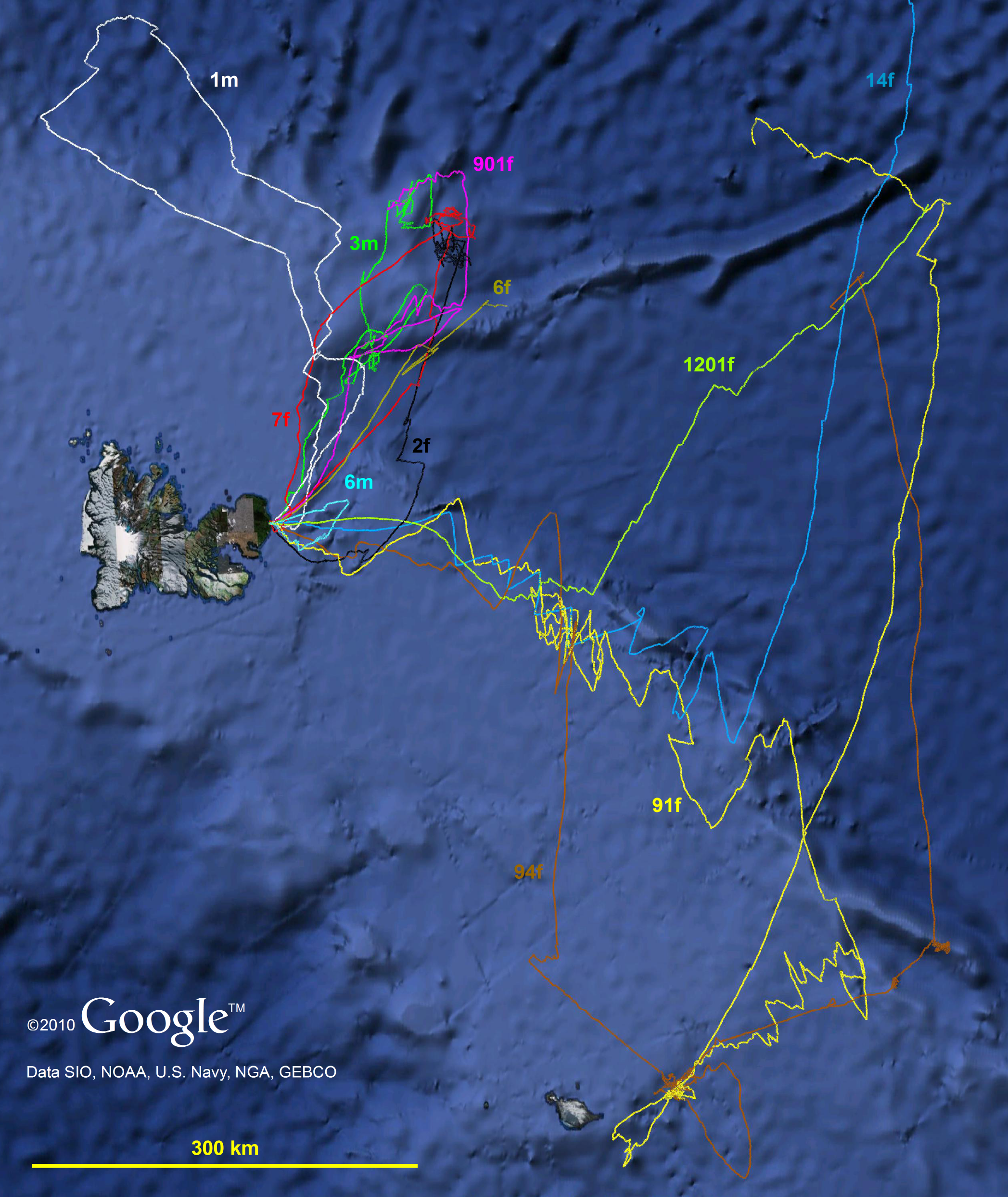

Supplement: Figure S4 — Albatrosses' long-distance flights. Individual paths (projected to the sea surface) of long-distance flights of 11 albatrosses are represented. Both males (1 m, 3 m, 6 m) and females (2f, 6f, 7f, 14f, 91f, 94f, 901f, 1201f) were tracked. Complete paths were recorded for 1 m, 6 m, and 7f individuals and information on the part of the paths is available for the other birds. The scope of the study was to obtain high resolution data, and not necessarily complete paths. Additional information on 14f path that was longer than others is provided in Fig. 1a. The recording times for the other five birds are short, and their paths are not presented. The image was generated in the Google Earth Pro software. (TIF) [file pone.0041449.s004.tif]
