# Supplementary material for: Flying at No Mechanical Energy Cost: Disclosing the Secret of Wandering Albatrosses
Source: PLoS One. 2012 Sep 5;7(9):e41449. doi: 10.1371/journal.pone.0041449 (PMC3434196; doi:10.1371/journal.pone.0041449)
Supplement: Text S1 — Calculation of fuel consumption of a gasoline engine producing the same power as a wandering albatross. (DOCX) [file pone.0041449.s005.docx]

**FUEL CONSUMPTION OF GASOLINE ENGINE PRODUCING THE SAME POWER AS A WANDERING ALBATROSS**

**Energy required by albatross**

Mass: **$m=8.5\text{ kg}$**

Drag-to-lift ratio: $C_{\text{D}}/C_{\text{L}}=0.05$

Speed: **$V=\text{70 km/h}$**

Energy required for $t_{\text{day}}=24\text{ h}$ flying: **$E_{\text{day}}=DVt_{\text{day}}=(C_{\text{D}}/C_{\text{L}})mgVt_{\text{day}}$**

Thus: **$E_{\text{day}}=7.0\times\text{ 10}^{6}\text{ J}$**

**Gasoline**

Energy: 4.0 10^7^ J/kg

Density: 0.75 kg/dm^3^

Efficiency: 25 %

Effective gasoline energy per litre: **$E_{\text{litre}}=7.5\times\text{ 10}^{6}\text{ J/dm}^{3}$**

**Fuel consumption per day**

$$m_{\text{fuel,day}}=E_{\text{day}}/E_{\text{litre}}$$

Thus: **$m_{\text{fuel,day}}=0.\text{93 dm}^{3}$**
