# Supplementary material for: Flying at No Mechanical Energy Cost: Disclosing the Secret of Wandering Albatrosses
Source: PLoS One. 2012 Sep 5;7(9):e41449. doi: 10.1371/journal.pone.0041449 (PMC3434196; doi:10.1371/journal.pone.0041449)
Supplement: Text S2 — Interactive 3-dimensional visualization of dynamic soaring. (DOCX) [file pone.0041449.s006.docx]

**INTERACTIVE 3-DIMENSIONAL VISUALIZATION OF DYNAMIC SOARING**

A .kmz file for Google Earth can be downloaded by following the link below. The file enables interactive visualization of the dynamic soaring cycle discussed in the article. This manoeuvre was reconstructed with high precision and 10 Hz resolution by means of GPS raw data post-processing. The file also holds approximately eleven minutes of flight preceding and following this manoeuvre. These data come from the coarse 1 Hz online solution provided directly by the GPS module.

**Instructions and hints for achieving the best visualization results**

- Open the file (Wandering Albatross Dynamic Soaring.kmz) by double clicking (MS Windows) or simply by dragging and dropping to Google Earth.
- Google Earth *sidebar*

If not already displayed when opening the file, activate the sidebar in the view menu. Enable or disable the various trajectory elements (described below) by clicking on the respective checkbox.

- Use of the *scroll wheel* of your mouse to navigate through the trajectory
  - Scroll the wheel for zooming
  - Press Ctrl and scroll for changing the map heading
  - *Press down the scroll wheel and move the mouse* for freely changing the camera angle.

**Displayed elements**

Click on the respective icons in the Google Earth map to display bubbles holding further information.

- **Dynamic soaring trajectory**

Eleven minutes of dynamic soaring. The purple arrows integrated in the trajectory indicate the flight direction. The displayed position fixes are the coarse solution calculated online by the GPS module with a rate of 1 Hz.

- **Dynamic soaring cycle**

Individual dynamic soaring manoeuvre as analysed in the article (Fig. 2a, 2b and 3). The 10 Hz position fixes come from GPS raw data post-processing.

- **Wind**

Blue arrows indicating wind direction according to NASA QuickSCAT L3 wind data.

- **Labels**

Display of additional information. Disable for unrestricted view using the respective sidebar checkbox.

- **Tour:** *Fly with the Albatross!*

Google Earth 5.0 and higher. Double click on the sidebar checkbox next to the camera symbol to take off.

If a previous version of Google Earth is installed on your machine, this element will not appear in the sidebar.

**Download the file here:**

[http://www.fsd.mw.tum.de/images/stories/Daten/Mitarbeiter/Traugott/Wandering Albatross Dynamic Soaring.kmz](http://www.fsd.mw.tum.de/images/stories/Daten/Mitarbeiter/Traugott/Wandering%20Albatross%20Dynamic%20Soaring.kmz)
