# Supplementary material for: Flying at No Mechanical Energy Cost: Disclosing the Secret of Wandering Albatrosses
Source: PLoS One. 2012 Sep 5;7(9):e41449. doi: 10.1371/journal.pone.0041449 (PMC3434196; doi:10.1371/journal.pone.0041449)
Supplement: Table S1 — Details on the recorded long-distance flights of tracked wandering albatrosses are given (see also Figure S4). The trip parameters presented were calculated based on segment of the trip for which GPS data were available. Travel speed reflects overall speed including flying and resting on the water surface. (DOCX) [file pone.0041449.s007.docx]

**Table S1|** Parameters of albatrosses’ long distance flights.

| № | Animal | Trip duration  (days) | Path length  (km) | Travel speed  (km/h) | Flight duration  (%) | Flight speed  (km/h) |
| --- | --- | --- | --- | --- | --- | --- |

| 1  2  3  4  5  6  7  8  9  10  11 | 6m  1201f  901f  6f  1m  7f  3m  2f  14f  94f  91f  **Mean±STD** | 0.82  0.93  0.95  0.99  3.55  3.80  4.16  4.38  6.02  6.38  6.47  **3.50±2.27** | 173.7  659.6  646.0  316.6  1115.7  765.7  842.5  746.7  4847.0  2348.8  3363.3  **1439±1471** | 8.84  29.62  28.25  13.38  13.09  8.40  8.43  7.09  33.52  15.34  21.67  **17.06±9.61** | 26.20  47.02  68.15  23.98  24.71  19.77  22.96  23.66  70.06  42.46  63.06  **39.27±19.81** | 53.30  76.12  56.81  61.31  64.29  57.02  54.10  50.41  61.21  57.37  55.57  **58.86±6.96** |
| --- | --- | --- | --- | --- | --- | --- |
